# Review of Mobile Apps for Women With Anxiety in Pregnancy: Maternity Care Professionals’ Guide to Locating and Assessing Anxiety Apps

**DOI:** 10.2196/31831

**Published:** 2022-03-23

**Authors:** Kerry Evans, Jasper Donelan, Stefan Rennick-Egglestone, Serena Cox, Yvonne Kuipers

**Affiliations:** 1 School of Health Sciences University of Nottingham Nottingham United Kingdom; 2 Digital Research University of Nottingham Nottingham United Kingdom; 3 School of Health Sciences Institute of Mental Health University of Nottingham Nottingham United Kingdom; 4 Edinburgh Napier University School of Health and Social Care Edinburgh United Kingdom

**Keywords:** anxiety, pregnancy, antenatal, mobile applications, digital interventions, mHealth, mobile app, psychological well-being, maternity, evaluation, quality assessment

## Abstract

**Background:**

Mental health and pregnancy apps are widely available and have the potential to improve health outcomes and enhance women’s experience of pregnancy. Women frequently access digital information throughout their pregnancy. However, health care providers and women have little information to guide them toward potentially helpful or effective apps.

**Objective:**

This review aimed to evaluate a methodology for systematically searching and reviewing commercially available apps that support pregnant women with symptoms of anxiety in order to assist maternity care professionals in identifying resources that they could recommend for these women.

**Methods:**

A stepwise systematic approach was used to identify, select, describe, and assess the most popular and highly user-rated apps available in the United Kingdom from January to March 2021. This included developing a script-based search strategy and search process, writing evaluation criteria, and conducting a narrative description and evaluation of the selected apps.

**Results:**

Useful search terms were identified, which included nonclinical, aspirational, and problem-based phrases. There were 39 apps selected for inclusion in the review. No apps specifically targeted women with anxiety in pregnancy. Of the 39 apps included in the review, 33 (85%) focused solely on mind-body techniques to promote relaxation, stress reduction, and psychological well-being. Only 8 of the 39 (21%) apps included in the review reported that health care professionals had contributed to app development and only 1/39 (3%) provided empirical evidence on the effectiveness and acceptability of the app. The top 12/39 (31%) apps were evaluated by 2 independent reviewers using the developed criteria and scores. There was a small negative correlation between the reviewers’ scores and app user rating scores, with higher user rating scores associated with lower reviewer scores.

**Conclusions:**

App developers, publishers, and maternity care professionals should seek advice from women with lived experience of anxiety symptoms in pregnancy to locate, promote, and optimize the visibility of apps for pregnant women. There is a lack of resources that provide coping strategies based on current evidence for the treatment of anxiety in pregnancy. Maternity care providers are limited in their ability to locate and recommend acceptable and trustworthy apps because of the lack of information on the evidence base, development, and testing of apps. Maternity care professionals and women need access to libraries of trusted apps that have been evaluated against relevant and established criteria.

## Introduction

Many pregnant women experience symptoms of anxiety; the prevalence of antenatal anxiety symptoms has been reported to be 13%-23% [1-3]. During the COVID-19 pandemic, the number of women with symptoms of anxiety in pregnancy has increased due to women’s concerns about virus transmission, accessing care, and social support [4,5]. Anxiety symptoms in pregnancy usually have similar affective and cognitive attributes to anxiety symptoms at other times [6], although concerns related to pregnancy may present as the predominant feature. Mild anxiety in pregnancy may be normal to prepare women for motherhood and protecting the fetus [6,7]. Anxiety symptoms become problematic when a significant amount of a woman’s time is consumed, when women are unable to redirect their focus to other tasks, or when everyday life and relationships are affected [6,8,9]. Antenatal anxiety is reported to be associated with postpartum depression, greater use of interventions during labor, reduced rates of breastfeeding, prematurity, and preterm birth [10-12].

The provision of web-based advice via mobile phones and the internet has been suggested to help reduce anxiety and stress in pregnant women [4,13]. Digitally delivered interventions are potential solutions to overcoming barriers to access treatment for perinatal mental health disorders. Interventions can be delivered as unguided resources to support or replace patient-provider interactions or as guided interventions that may include live interactions over telephone or video or contact with therapists using digital messaging [14]. Increasing midwives’ and maternity care providers’ awareness of digitally delivered information and supportive interventions could assist in signposting pregnant women to effective resources [15]. During pregnancy, women frequently access digital information [16], although information accessed through web-based sources is rarely discussed with health care providers and the providers themselves may be unaware of web-based information and its accuracy [17]. Mobile apps have the potential to positively influence health behavior and health outcomes [18,19] and enhance women’s experience of maternity care and pregnancy [20]. The availability of mobile apps has increased significantly over the past few years. There are currently more than 400,000 health apps available from Google Play [21] and the App Store [22]; however, there are fewer than 10,000 downloads for many of these apps and 25% are never used after installation [18].

Mental health and pregnancy apps are widely available; however, health care providers and women have little information on which apps may be helpful or which to avoid because these are ineffective or have potentially harmful content [23]. Platforms such as the NHS Apps Library [24] have only recently been developed to assist patients and the public in finding trusted health and well-being apps and include general pregnancy and mental health apps [25]. User ratings presented by the app stores, on the other hand, do not provide a measure of clinical appropriateness, safety, or efficacy, and the availability of clinical data to guide app recommendations is poor [23]. Moreover, the health app industry is commercially dominated and lacks regulation [16].

Health apps for use in pregnancy or to support individuals with anxiety symptoms have been assessed as having poor quality [26-28], lacking evidence-based content [29], and being ineffective or potentially harmful [30]. Other reviews of pregnancy apps have reported that apps contained little or no pregnancy-specific information [26] and contained information that was potentially harmful for pregnant women [31]. Health professionals and app users have reported a preference for using pregnancy apps that are relevant to their local health care context and come from a trusted source. There is a need for greater health professional engagement in app development and increased awareness of and guidance for use of these resources [16].

Several methodologies have been proposed for evaluating the quality of apps [23,25], but the validity of app rating measures is not yet established [18] and standardized measures with high interrater reliability are required. Nouri et al [18] conducted a systematic review of existing health app assessment tools (N=23). In total, there were 38 main classes of assessment criteria, which the reviewers arranged into 7 main criteria: Design, Information/Content, Usability, Functionality, Ethical Issues, Security and Privacy, and User-perceived value. Powell et al [23] evaluated the interrater reliability of existing app quality measures, but only criteria for “App interactiveness” and “feedback” reached the threshold for agreement, with items related to security and privacy, number of ratings, research base, authorship, attribution, and product advisory support reaching near threshold levels of agreement (α levels of .5 or more). Items with low interrater reliability (α .3 or less) included more subjective measures, including perceived and claimed effectiveness, ease of use, errors, and performance issues. The authors state that even more objective measures can be missed by a reviewer and suggest that the evidence base of the app may be a more reliable indicator of effectiveness. Therefore, clinicians need to review apps personally before making recommendations, discuss apps with colleagues and service users, and apply clinical judgment [23].

The purpose of this review was to evaluate a methodology to systematically search and review commercially available apps to support pregnant women with symptoms of anxiety. The review focused on identifying app resources that could be used to complement maternity and perinatal mental health care and identifying methods of searching and evaluation that can be adopted by maternity care professionals with limited time and resources.

## Methods

This review adopted a stepwise systematic approach to identify, select, describe, and assess the most popular and highly user-rated apps available in the United Kingdom from January to March 2021. The review team included individuals with expertise in maternity care and research, mental health care, and digital research and a lived experience of maternity. Ethics approval was not required for this review because primary data were not collected.

### Developing an App Search Strategy

The first stage involved identifying key search words and terms (Table 1). Research and academic keywords and Medical Subject Headings terms (eg, Anxiety AND Pregnancy) were piloted on Android (Google Play, Google) and iOS (App Store, Apple). These search terms led to limited results and did not locate preidentified “marker” apps (Mind the Bump [32] and Headspace [33]). The Headspace and Mind the Bump apps were selected as marker apps because they are recommended by the National Health Service or established mental health charities. General phrases were brainstormed by the review team, considering terms that women may use to search for resources as well as descriptive and marketing terms that app developers might employ. The search strategy also considered the work by Wexler et al [34] in a study related to our own. Wexler et al [34] analyzed data from pregnancy social media forums. They found that the frequency of word appearance in relation to other words revealed clusters of words that have a high probability of appearing together around certain topics. Therefore, word clusters for anxiety about pregnancy and labor were included in the search terms. Members of an established public involvement group also contributed by identifying words they would use to locate information and resources. Service users highlighted that women may also search for specific pregnancy-related aspects that cause anxiety (eg, miscarriage, giving birth) or use words to describe how they would like to feel (eg, calm, stress-free, relaxed). Again, these were included in the key search words and terms for the present review.

Apps were included in the review if they were available in English, aimed at or referred to use in pregnancy, and included advice, information, exercises, or techniques to improve symptoms of anxiety, worry, fear, or stress. Exclusion criteria were anxiety apps that did not reference pregnancy in the app information and pregnancy apps that did not reference anxiety, worry, stress, fear, or emotional or mental health.

**Table 1:** Developing search terms for Google Play and the App Store.

Research literature searching keywords	Web-based words and phrases
Pregnancy, antenatal, perinatal, childbearing	Pregnant, pregnancy, motherhood, mother (mum, mom, mama, momma), baby, birth (childbirth), miscarriage, movements (fetal/baby movements), labor, maternity
Anxiety	Anxious (anxiety), worry (worries), concerns; stress, distress; ear, panic, scared, nervous; mind, emotion, thoughts, mood; mental health; therapy (CBT^a^); relax, calm; cope (coping); help, care, relief, cure; wellbeing

^a^CBT: cognitive behavioral therapy.

### Search Process

To identify efficient and effective search terms that could be used by maternity care professionals, previously developed scripts were used to search the UK version of Google Play and the App Store. The search was conducted from January to March 2021 using the script-based approach developed by Stawarz et al [35]. The scripts were modified using alternative combinations of the suggested keywords. Each combination of keywords and the number of apps located are shown in Multimedia Appendix 1. Search results included the app name, short description, rating, and developer’s details. After script-based searching, a simple web-based keyword search of Google Play and the App Store was performed to compare the search results with those of script-based searching and to locate additional apps. Changing the country code (ie, United Kingdom, United States) did not appear to impact the search result. Changing the word order resulted in different search results on Google Play but not on the App Store. Manual screening of the app search results was completed by evaluating the app title and description against the inclusion and exclusion criteria.

### Identifying Evaluation Criteria for the Apps

Evaluation criteria that considered the findings from existing pregnancy and mental health app reviews were selected. The criteria were therapeutic or supportive content (reflective of the evidence base or clinical standards) [29,35,36], relevance for a pregnant population [26,31,37], and compliance with existing app quality measures (Multimedia Appendix 2). The following therapeutic or supportive content was evaluated against review-level evidence and clinical recommendations for perinatal and common mental health disorders [38-41]:

1. Psychoeducation: Aids in the identification of anxiety disorders and improves understanding and treatment options for anxiety.

2. Low-intensity psychological interventions including individual nonfacilitated, guided self-help or psychoeducational group. Includes written or electronic materials based on the treatment principles of cognitive behavioral therapy (CBT).

3. Mind-body interventions such as yoga or hypnotherapy.

4. Referral and signposting to services for women requiring specialist diagnosis and care.

It is important to consider whether the current evidence for an app is sufficient or relevant for a particular population [37]. Therefore, the following questions were developed by the reviewers to identify the availability of information that women could search for [42].

1. How do I know if I have anxiety?

2. How do I know the severity of my anxiety symptoms?

3. Where can I go for help for my anxiety in pregnancy?

4. How can I access specialist help for my anxiety in pregnancy?

5. What are the treatment options for anxiety during pregnancy?

6. What can I do to help my anxiety symptoms?

Based on the review by Nouri et al [18], quality criteria were selected to meet the review objectives. Subjective measures of app quality have been reported as having low interrater reliability [23]. Quality criteria were therefore selected that focused on objective measures and minimized the number of subjective responses. Efforts to reduce the subjective measures also considered end-user preference. Criteria identified by health care professionals as important may not reflect women’s needs and preferences, as different individuals may find a particular app more or less useful depending on their personal needs, circumstances, experiences, and education level [25,43]. User ratings as reported in Google Play and the App Store were also presented alongside reviewers’ evaluation scores for comparison.

### Analysis

Descriptive data were collected (where available) for all apps included in the review. This included the app developer and country, number of downloads, number of ratings, rating score, version number, costs, and date of the last update. Narrative description and evaluation of the highest scoring, free to download, and most popular apps were completed [29]. Apps were identified based on the app download numbers and user rating (0-5 stars) provided by Google Play and the App Store: more than 10,000 installs, a star rating of 3.5 or higher, and free to download in Google Play; and more than 10 ratings, a star rating of 3.5 or higher, and free to download in the App Store.

This resulted in the selection of 12 apps that were then evaluated by 2 independent reviewers using the criteria presented in Multimedia Appendix 2. The extent to which the app addressed the criteria was scored on a 2-point scale (0, information is absent/negative response; 1, information is present/positive response). The overall score was calculated and interpreted with caution because of the lack of validity and reliability of app quality measures [23]. Scores were used to assist in interpreting the review findings and to generate discussion on the mechanisms for selecting and recommending potentially beneficial apps (Table 2).

**Table 2:** Quality scores of the highest user rated and most popular apps in the review.

App name	Maternity and mental health policy and evidence base score (out of 6)	General app features quality score (out of 8)	Combined score (out of 14)	User rating score from Google play/App store (out of 5)
Antenatal Yoga, Meditation + Education (YogiBirth)	1	3	4	4.3/4.7
Baby Buddy	4	7	11	2.8/4
Carry: Pregnancy Workouts	5	4	9	—/4.7
Hypnobirthing: Calm Birth	1	3	4	—/4.4
Hypnobirthing - Pregnancy, Music & Tracker	1	3	4	3.4/—
IHypnobirth	1	4	5	—/3.8
Keleya: Pregnancy Fitness & Tracker + Baby Due Date	1	5	6	4.1/—
Music for Pregnancy Relaxation	0	3	3	4.2/—
Pregnancy Care Tips	0	1	1	3.6/—
Pregnancy Music Collection 200	0	2	2	4.4/—
Pregnancy Yoga Exercises	1	4	5	4.0/—
Pregnancy Yoga Exercises – Prenatal Yoga	1	3	4	3.8/—

## Results

### Apps Located and Included in the Review

**Figure 1:**
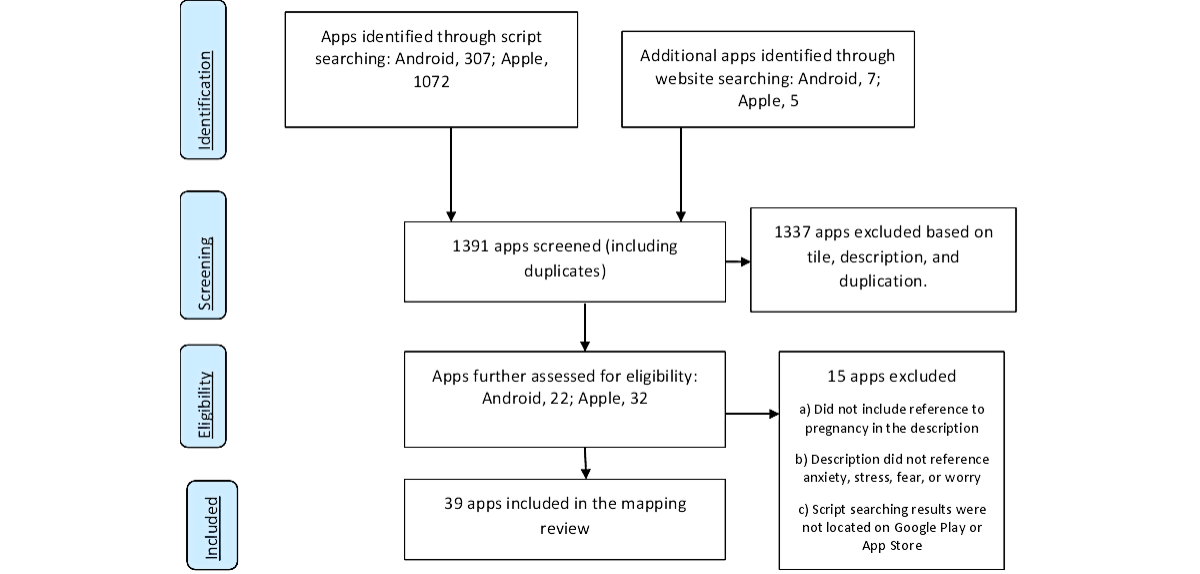
Adapted PRISMA diagram: anxiety apps for pregnant women. PRISMA: Preferred Reporting Items for Systematic Reviews and Meta-Analyses.

In total, 1391 apps were located, and 1337 (96%) apps were subsequently excluded based on the title, description, and duplication (Figure 1). A total of 39/1391 (3%) apps were included in the review (Multimedia Appendix 3).

**Figure 2:**
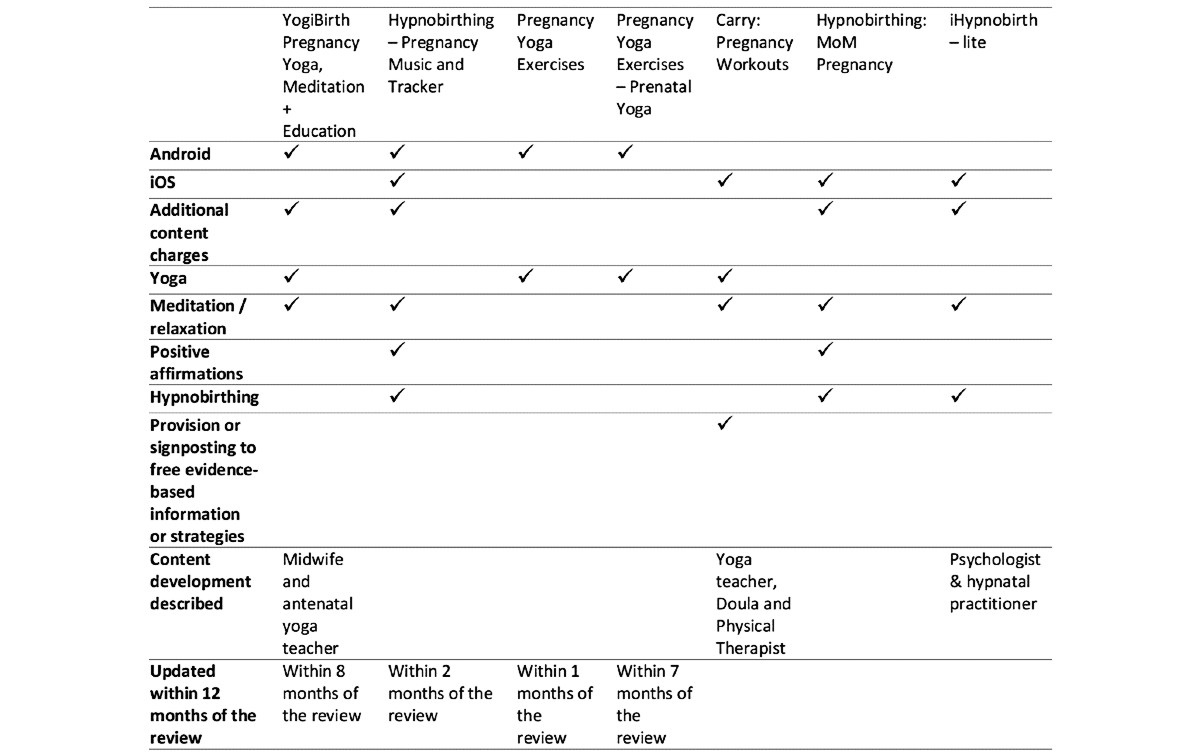
Yoga, relaxation and hypnobirthing apps.

No apps specifically targeted women with anxiety in pregnancy. Various apps focused on providing well-being support during pregnancy and included stress relief, relaxation, and mental health advice as part of a general approach to well-being in pregnancy. Of the 39 apps included in the initial review, 33 (85%) focused solely on mind-body techniques to promote relaxation, stress reduction, and psychological well-being. Mind-body techniques included relaxation, mindfulness, hypnosis, yoga, positive affirmations, and meditation. A total of 3/39 (8%) apps provided informational support, of which 1 (33%) focused on psychoeducation and 2 (67%) were multicomponent and provided information support, cognitive, or mind-body techniques. Only 8 of the 39 (21%) apps included in the review reported that health care professionals had contributed to app development (Figure 2). Only 1/39 (3%) app (Baby Buddy) provided empirical evidence on the effectiveness and acceptability of the app. 

There was a small negative correlation between the reviewers’ scores and the user rating scores (r=−0.27; 12/39, 31%; *P*=.39) with higher user rating scores associated with lower reviewer scores.

### Description of the Apps Included in the Evaluation

#### Yoga, Relaxation, Meditation, and Hypnobirthing Apps

Of the 12 apps, 7 (58%) provided elements of relaxation, hypnobirthing, yoga, and relaxation (Figure 2) [44-50]. Of the 7 apps, 4 (57%) provided exercises to improve mood, anxiety coping, or mental well-being [44-47], although no supporting evidence or information about anxiety or mental well-being was provided. Only 1/7 (14%) app [48] included information about depression and anxiety in pregnancy and the postnatal period via links to national health care websites and helplines for factsheets.

#### Music Apps

Of the 12 apps, 2 (17%) provided music for relaxation in pregnancy and were very similar in content and appearance: Music for Pregnancy Relaxation [51] and Pregnancy Music Collection [52]. Updates had not been completed within 12 months of the review. Both apps reported that music could reduce stress and anxiety in pregnancy and support fetal brain development. No empirical evidence was provided to support the claims. Both apps provided recordings of classical music or soothing sounds through a simple interface.

#### Informative Apps

Pregnancy Care Tips [53] was available on Android and iOS devices, although it had not been updated within 5 years of the review. At the time of publication, the app was no longer available to download. The app provided written “tips” and information on pregnancy symptoms, diet, exercise, and maternity care. The information appeared to have been translated into English and certain phrases and terms were difficult to understand and not reflective of UK maternity care procedures. No information was provided on the development of the app, professional input, or supporting evidence.

Keleya: Pregnancy Fitness & Tracker + Baby Due Date [54] was available on Android and iOS devices. Keleya was promoted as an “All-in-One App” for pregnancy and contains yoga exercises, nutritional advice, and information on the progress and symptoms of pregnancy. Content was provided in written and audio material, which could be personalized by responding to user input and detailing stage of pregnancy, goals, and symptoms. The app reported reducing anxiety through meditation exercises. No empirical evidence was provided, and there was no information on app development or health care professional input. Free content was very limited, and additional features such as social media community access, information, and exercises required a subscription fee. Information on anxiety in pregnancy was limited to user input to track anxiety symptoms with no further information, support, or signposting available.

Baby Buddy [55] is a National Health Service–endorsed app from the charity Best Beginnings. The app was updated within 1 month of the review and is free on Android and IOS devices. A website link from the app provided links to evaluation and impact studies within UK maternity care institutions. The app reported guiding women through pregnancy by providing information through written and video materials. Baby Buddy provided numerous video clips on the topic of anxiety and depression, although there were no specific resources to help women develop coping strategies and techniques to manage symptoms of anxiety. The information included an overview of anxiety disorders, CBT approaches, how or when to seek help with mental health, and the benefits of peer support. Baby Buddy reported that the text and video clips were quality-assured by professional Royal Colleges and other health organizations. The app was interactive and enabled women to make notes of questions to ask their midwife and input information to access personalized content. Signposting was provided for local maternity services and support groups.

## Discussion

### Locating and Evaluating Apps

The purpose of this review to evaluate a methodology to systematically search for and review commercially available apps was addressed by identifying useful search terms that were nonclinical and included aspirational phrases as suggested by the study service user group. The terms included “calm,” “relax,” “relief,” “cope,” and “well-being” as well as some problem-based phrases such as “stress,” “fear,” and “anxiety.” However, no clear recommendations were identified for the searching of apps to help maternity care professionals identify useful resources.

The evaluation components developed for the review provided a useful framework for maternity care professionals to assess the therapeutic or supportive content in the context of pregnancy. The quality criteria focused on more objective measures and aimed to minimize the number of subjective responses. However, the binary coding (1, information/component present; 0, information/component absent) was not amenable to capture criteria that were partly addressed by the app or where particular types of content were presented differently within the app [42,56]. The quality criteria have been revised in response to the findings and are presented in Multimedia Appendix 4. Furter studies to evaluate the reliability of the criteria are required [18].

Many of the apps that purported to reduce anxiety symptoms did not include any link to peer-reviewed literature or the evidence base. Other reviews have reported the lack of provision of evidence-based app content [28,35,36,56]. Although web-based mindfulness and CBT approaches have been reported as effective in reducing anxiety and other mental health concerns in perinatal populations [57,58], evidence-based information was not presented in the apps. The lack of clearly defined content with links to the evidence base may hinder the ability of maternity care professionals to determine the quality of the app [56]. However, this review highlighted that only 1 of the 7 (14%) most popular and highly rated mind-body apps provided any rationale for the approach or links to the evidence base.

The lack of correlation between the presence of evidence-based information or strategies and the popularity of mental health apps has been highlighted in recent reviews [35,56,59]. This suggests that features other than the provision of evidence-based information are important to and valued by women [35]. To further highlight this point, this review has demonstrated that the quality evaluation scores assigned by the review team (health care professionals and researchers) did not reflect user ratings (displayed by the app platforms). Because of the lack of information on the development of the apps, it is difficult to know whether women’s views were accessed or whether the design and content of the app reflected women’s needs.

User experience and engagement are important factors in the overall effectiveness of apps [35]. Stawarz et al [35] identified that interactive features and customization were important in improving user engagement with CBT for depression. Positivity (ability to capture positive and negative thoughts) along with privacy, security, and trust was associated with improved user experience. For this review, engagement with service users also highlighted that women may wish to use “positive” and aspirational words to describe how they would like to feel (eg, calm, stress-free, relaxed) when they search for potentially useful apps. Researchers should aim to make mobile health interventions with evidence-based treatments attractive and accessible and focus on user desirability and experience early in the design phase [59]. A combination of approaches to maximize user engagement and experience with evidence-based strategies is required to deliver potentially effective app-based strategies to support women with anxiety in pregnancy.

Pregnant women may benefit from remotely delivered interventions to help them cope with symptoms of anxiety if they are provided with web-based contact with a health care professional or peer community and may be more motivated to complete interventions that are perceived as relevant or tailored to their needs and situations [60]. Only 2 of the 39 (5%) apps included in the review provided a psychological therapeutic approach, with most apps providing mind-body techniques and exercises to support women’s general well-being throughout pregnancy, labor, and birth. Only 1/39 (3%) app was endorsed by a health care organization (Baby Buddy). Although it did not provide any therapeutic content, this app did provide information and signposting. Apps that offered coping strategies for women with symptoms of anxiety had very little information about how or when to seek help or signposting to supportive services.

The apps included in this review were promoted as either general pregnancy well-being apps (including diet, exercise, fetal growth and well-being, labor, and birth) or general relaxation apps. This review did not locate any apps that were solely focused on anxiety symptoms in pregnancy. A systematic review of perinatal web-based psychological treatments for clinical levels of maternal anxiety and depression [61] also did not locate any interventions targeted to the reduction of anxiety disorders or comorbid depression and anxiety. Although no interventions were tailored to anxiety or recruited women with a diagnosed anxiety disorder, the pooled analysis demonstrated medium and significant group differences favoring web-based interventions over control conditions for anxiety outcome measures. The authors of this previous systematic review recommend that interventions be specifically developed for this neglected area of perinatal mental health care.

The perceived reliability and trustworthiness of web-based pregnancy information have been reported to increase in women when the resource is regularly updated and when it is recommended by a health care professional [62]. Involvement of the health care provider has been reported to help individuals understand what apps can and cannot do [29]. Evaluation of app quality by health care professionals should not be a substitute for women’s preferences, usability, and other personal factors necessary for selecting an app [29]. Zelmer et al [63] sought consensus from a broad group of stakeholders on guiding principles and criteria for a framework to assess e-mental health apps. The resulting principles are similar to the criteria developed for this review across categories of security, usability, evidence base, and functionality. It would not be possible for most maternity care professionals to assess the fast-growing health apps market. Access to technologies, curated compilations, or libraries of effective apps would, however, help maternity care professionals feel more confident in recommending and signposting women to potentially beneficial resources [25,64]. Platforms such as the NHS Apps Library have started to develop such resources, inviting developers to submit apps for assessment of regulatory, clinical, security, and technological criteria [65].

### Study Strengths and Limitations

For this review, potentially useful criteria for rating the quality of the content and the function of apps were suggested to support women with symptoms of anxiety in pregnancy. The criteria were based on previous research, and validity and reliability testing was not conducted nor was it within the remit of the study. The security and privacy policies of each app were not fully scrutinized, although an assessment of whether security and privacy policies were reported in the app information was included. The American Psychiatric Association has developed a framework for app evaluation [37], which begins with the assessment of compliance with safety and privacy criteria. These criteria must be met before the evaluation continues to assess benefit and efficacy, engagement, and data sharing. Simply checking for the existence (or absence) of a privacy policy will help identify questionable apps [37].

### Conclusions

Locating potentially useful apps is not a straightforward process and requires a different approach to that used in traditional academic search. Keywords that reflect women’s search queries and that can help women and maternity care providers navigate app libraries need to be developed. App developers, publishers, and maternity care professionals should seek advice from women with lived experience of anxiety symptoms in pregnancy to locate, promote, and optimize the visibility of apps for a diverse population of pregnant women. This review did not locate any resources that provided coping strategies or therapeutic approaches for anxiety that were based on the current evidence base for the treatment of anxiety in pregnancy. The rationale, development, and testing of apps included in this review were underreported, which may hinder the ability of maternity care providers to easily locate useful, acceptable, and trustworthy resources. Potentially useful quality criteria have been presented, which require further development and testing. Maternity care professionals should be aware that features of apps other than the provision of evidence-based information and approaches are important to app users. Features such as interactivity and customization may improve user engagement, and positive framing using aspirational statements may attract women with symptoms of anxiety when they look to select an app resource. Maternity care professionals and women would benefit from access to libraries of trusted apps that have been evaluated against relevant and standardized criteria.

## Appendix

Multimedia Appendix 1 Keyword results in the search strategy.

Multimedia Appendix 2 Suggested criteria for evaluating the quality of apps for women with anxiety in pregnancy.

Multimedia Appendix 3 Included apps from Google Play and App Store (information accessed March 4, 2021).

Multimedia Appendix 4 Evaluation criteria for pregnancy anxiety apps (adapted from Van Singer et al [42] and Nouri et al [18]).

## Abbreviations

CBT: cognitive behavioral therapy
