# Supplementary material for: Review of Mobile Apps for Women With Anxiety in Pregnancy: Maternity Care Professionals’ Guide to Locating and Assessing Anxiety Apps
Source: J Med Internet Res. 2022 Mar 23;24(3):e31831. doi: 10.2196/31831 (PMC8987965; doi:10.2196/31831)
Supplement: Multimedia Appendix 1 [file jmir_v24i3e31831_app1.docx]

**Multimedia Appendix 1.** Keyword results in the search strategy.

|  | No results found |
| --- | --- |
|  | No relevant Apps found |
|  | 1-5 potentially relevant App found |
|  | 6-10 potentially relevant Apps found |
|  | 11-20 potentially relevant Apps found |
|  | > 20 potentially relevant Apps found |

|  | Google Play  Android | App store  iOS |
| --- | --- | --- |
| Anxiety+pregnancy |  |  |
| Anxiety+motherhood |  |  |
| Baby+moving [movements] |  |  |
| Birth+stress |  |  |
| Birth+wellbeing |  |  |
| Calm+pregnancy |  |  |
| Care+pregnancy |  |  |
| Childbirth[birth]+fear |  |  |
| Concerns+pregnancy |  |  |
| Cope [coping]+pregnancy |  |  |
| Cope [coping]+[mama/mom/momma/mum/motherhood] |  |  |
| Distress+pregnancy |  |  |
| Emotion[s]+pregnancy |  |  |
| Fear+childbirth[birth] |  |  |
| Fear+labour |  |  |
| Fear+pregnancy |  |  |
| Help+pregnancy |  |  |
| Mama[momma/mum/mom]+mind |  |  |
| Mama[momma/mum/mom]+mood |  |  |
| Mama[momma/mum/mom]+stress |  |  |
| Mama[momma/mum/mom]+worry[worries] |  |  |
| Maternity+worry |  |  |
| Mental health+pregnany |  |  |
| Mind+bump |  |  |
| Mind+[mama/mom/momma/mum/motherhood] |  |  |
| Mind+pregnancy |  |  |
| Mindfulness+pregnancy |  |  |
| Miscarriage |  |  |
| Mom+[coping/mind/mood/stress/worries/worry |  |  |
| Momma+[coping/mind/mood/stress/worries/worry |  |  |
| Mood+[mama/mom/momma/mum/motherhood] |  |  |
| Mood+pregnancy |  |  |
| Motherhood+coping |  |  |
| Motherhood+[anxiety/stress/worry] |  |  |
| Normal+pregnancy |  |  |
| Pain+pregnancy |  |  |
| Panic+pregnancy |  |  |
| Pregnany+anxiety |  |  |
| Pregnany+care |  |  |
| Pregnancy+cbt[emotion/mind/nervous/therapy] |  |  |
| Pregnancy+concerns |  |  |
| Pregnancy+coping[cope] |  |  |
| Pregnancy+cure |  |  |
| Pregnancy+fear |  |  |
| Pregnancy+mental health |  |  |
| Pregnancy+mood |  |  |
| Pregnancy+relax[relaxation] |  |  |
| Pregnancy+relief |  |  |
| Pregnancy+stress |  |  |
| Pregnancy+wellbeing |  |  |
| Relaxation+pregnancy |  |  |
| Stress+[mama/mom/momma/mum] |  |  |
| Stress+pregnancy |  |  |
| Therapy+pregnancy |  |  |
| Thoughts+pregnancy |  |  |
| Wellbeing+pregnancy |  |  |
| Worries[worry]+pregnancy |  |  |
| Worries[worry]+ [mama/mom/momma/mum] |  |  |
