# Supplementary material for: Review of Mobile Apps for Women With Anxiety in Pregnancy: Maternity Care Professionals’ Guide to Locating and Assessing Anxiety Apps
Source: J Med Internet Res. 2022 Mar 23;24(3):e31831. doi: 10.2196/31831 (PMC8987965; doi:10.2196/31831)
Supplement: Multimedia Appendix 2 [file jmir_v24i3e31831_app2.docx]

**Multimedia Appendix 2.** Suggested criteria for evaluating the quality of apps for women with anxiety in pregnancy.

| Maternity and perinatal mental health policy and evidence base - *adapted from Van Singer et al. [42]* | | |
| --- | --- | --- |
|  | Information is present = 1 | Information is absent = 0 |
| How do I know if I have anxiety? | | |
| 1. Psychoeducation of anxiety symptoms: App presents comprehensive information about anxiety in the context of pregnancy including symptoms and types of disorders. |  |  |
| How do I know the severity of my anxiety symptoms? | | |
| 2. App provides women with access to established validated self-assessment tools with advice or signposting depending on the results |  |  |
| Where can I go for help? | | |
| 3. App includes advice about when and how to contact midwife, GP or other maternity care professional. Provides contact information and ways to open discussions |  |  |
| How can I access specialist help? | | |
| 4. App includes information about supportive Perinatal Mental Health Services with referral pathways or contact information |  |  |
| What are the treatment options for anxiety? | | |
| 5. App provides women with an overview of options for the treatment for anxiety in pregnancy |  |  |
| What can I do to help my anxiety symptoms? | | |
| 6. App presents a single or multiple evidence-based coping strategies for anxiety in pregnancy |  |  |
| App quality assessment based on published assessment measures – *adapted from Nouri et al. [18]* | | |
| Design and useability | | |
|  | Yes = 1 | No = 0 |
| 7. Is the App easy to read and well organised? |  |  |
| 8. Is the App is available in other languages? |  |  |
| 9. Is the easy App to use? |  |  |
| Information and content | | |
| 10. Are sources of information and references included with distinction between scientific and non-scientific content? |  |  |
| 11. Is the information in the App objective and free from bias (the App is not selling additional features or products)? |  |  |
| 12. Has the App been trialled or tested and reported in published literature? |  |  |
| 13. Has the App been updated in the past 6 months? |  |  |
| Security and privacy | | |
| 14. Does the App provide clear information about security and privacy policies related to personal health information? |  |  |
