# Supplementary material for: Review of Mobile Apps for Women With Anxiety in Pregnancy: Maternity Care Professionals’ Guide to Locating and Assessing Anxiety Apps
Source: J Med Internet Res. 2022 Mar 23;24(3):e31831. doi: 10.2196/31831 (PMC8987965; doi:10.2196/31831)
Supplement: Multimedia Appendix 3 [file jmir_v24i3e31831_app3.docx]

**Multimedia Appendix 3.** Included apps from Google Play and App Store (information accessed March 4, 2021).

| App name | Platform | Located via script, webpage or both | Main supportive features to support anxiety symptoms in pregnancy | Media | Developer  *Country* | Number of reviews / Installs | Professional input in the App content | Pricing |
| --- | --- | --- | --- | --- | --- | --- | --- | --- |
| Antenatal Yoga, Meditation + Education: YogiBirth | Google Play | Script & webpage | Mind-body: Yoga | Audio, video and written information | Yogibirth  *Australia* | 288 reviews  10,000+ installs | Created by a midwife | Free with in-app purchases |
| Baby Buddy - Pregnancy, birth & baby support | Google Play | Store webpage | Informational support | Videos and written information | Best Beginnings  *UK* | 599 reviews  100,000+ installs | NHS endorsed | Free |
| Calm Birth | Google Play | Store webpage | Mind-body: meditation | Video, audio and written information | Anna Humphreys  *US* | 10 reviews  1,000+ installs | Not reported | Free |
| Expectful Meditation & Sleep | Google Play | Store webpage | Mind body: meditation | Audio and written information | Expectful  *US* | 200 reviews  10,000+ installs | Not reported | $9.99 monthly |
| Expecting: positive pregnancy with affirmations | Google Play | Script & webpage | Mind body: meditation and mindfulness | Audio and written information | Leverage IQ & Sami Apps  *Spain* | 63 reviews  10,000+ installs |  | Free with in-app products |
| GentleBirth Hypnobirthing | Google Play | Store webpage | Mind body: meditation and mindfulness | Audio and written information | Positive Birth App Development  *US* | 341 reviews  10,000+ installs | Not reported | $12.99 monthly |
| Hypnobirthing - Pregnancy, Music & Tracker | Google Play | Script & webpage | Mind body: meditation and mindfulness | Audio and written information | Derby Beenken *US* | 106 reviews  10,000 downloads | Not reported | Free with in-app purchases |
| Headspace: Meditation & Sleep  Pregnancy pack | Google Play | Script & webpage | Mind body: meditation and mindfulness | Audio and written information | Headspace  *US* | 190,657 reviews  10,000,000+ installs | Not reported | £5.99 monthly |
| Mindful Mamas: Meditation, Calm, & Mindfulness | Google Play | Script & webpage | Mind body: meditation and mindfulness | Audio and written information | Mindful Mamas Club  *US* | 106 reviews  50,000+ installs | Not reported | $9.99 monthly |
| Mind the bump | Google Play | Script & webpage | Mind body: mindfulness | Audio and written information | Smiling mind  *Australia* | 195 reviews  10,000+ installs | Written by psychologists and psychiatrists | Free |
| MindFeed App - Mindfulness & Meditation | Google Play | Script | Mind body: meditation and mindfulness | Video, audio and written information | Self-development sciences  *Not reported* | 17 reviews  100+ installs | Not reported | $4.76 month |
| Music for pregnancy relaxation | Google Play | Store webpage | Mind body: relaxation | Audio | Ultimate mobile  *Vietnam* | 4,593 reviews  1,000,000+ installs | Not reported | Free |
| Keleya- Pregnancy & Baby App | Google Play | Store webpage | Mind body: relaxation  Informational support | Video, audio and written information | Keleya Digital-Health Solutions GmbH  *Germany* | 303 reviews  10,000+ installs | Not reported | Free version with premium subscription options |
| Positive Pregnancy with Andrew Johnson | Google Play | Script & webpage | Mind body: meditation | Audio and written information | Universal relaxation *UK* | 19 reviews  1,000+ installs | Not reported | £2.39 |
| App name | Platform | Located via script, webpage or both | Main supportive features to support anxiety symptoms in pregnancy | Media | Developer  *Country* | Number of reviews / Installs | Professional input in the App content | Pricing |
| Pregnancy care tips | Google Play | Script | Informational support | Written information | EST  *India* | 284 reviews  50,000+ installs | Not reported | Free |
| Pregnancy music collection 200 | Google Play | Script | Mind body: relaxation | Audio | Tiaoz hisoft  *Not reported* | 1,113 reviews  100,000+ installs | Not reported | Free |
| Pregnancy Yoga Exercises | Google Play | Store webpage | Mind body: yoga | Video, audio and written information | Home fitness  *Not reported* | 536 reviews  100,000+ installs | Not reported | Free |
| Pregnancy Yoga Exercises – Prenatal Yoga | Google Play | Store webpage | Mind body: yoga | Video, audio and written information | Free education app *India* | 54 reviews  10,000+ installs | Not reported | Free |
| Prenatal Yoga poses | Google Play | Script & webpage | Mind body: yoga | Video, audio and written information | Stay fit with Samantha *Netherlands* | 35 reviews  10,000+ installs | Not reported | Free with in-app products |
| Silatha: Self-love & Meditation for women and mums | Google Play | Script | Mind body: meditation | Audio and written information | Silatha: Mindfulness, Balance, Selflove Meditation *UK* | 32 reviews  5,000+ installs | Not reported | In-app purchases |
| Antenatal Yoga, Meditation + Education YogiBirth | App store | Store webpage | Mind-body: Yoga | Videos and written information | Yogibirth *Australia* | 170 ratings | Created by a midwife | Free with in-app purchases |
| Babybuddy | App store | Store webpage | Informational support | Video, audio and written information | Best beginnings *UK* | 173 ratings | NHS endorsed | Free |
| Calm birth | App store | Script & webpage | Mind-body: meditation | Video, audio and written information | Anna Humphreys  *US* | NR | Not reported | Free |
| Calm Childbirth and Hypnobirthing | App store | Script & webpage | Mind-body: meditation and hypnosis | Audio and written information | Aluna Moon  *Not reported* | 7 ratings | Not reported | Free with in-app products |
| Carry prenatal yoga meditation | App store | Script & webpage | Mind-body: meditation | Audio and written information | Carry  *US* | 25,161 ratings | Not reported | Free with in-app products |
| Expectful meditation sleep | App store | Store webpage | Mind body: meditation | Audio and written information | Expectful  *US* | 263 ratings | Not reported | $9.99 monthly |
| Gentlebirth hypnobirthing | App store | Script & webpage | Mind body: meditation and mindfulness | Audio and written information | Positive Birth App Development  *US* | 13 ratings | Not reported | $12.99 monthly |
| Hypnobirthing: Calm Birth App | App store | Script & webpage | Mind body: meditation and mindfulness | Audio and written information | ATN  *Not reported* | 26 ratings | Not reported | Free with in-app products |
| IHypnobirth | App store | Store webpage | Mind body: meditation, hypnosis and mindfulness | Audio and written information | Charlotte Maslen *US* | 39 ratings | Psychologist | £3.99 with free lite version |
| Joyful pregnancy Glenn Harrold | App store | Script & webpage | Mind body: hypnosis and relaxation | Audio and written information | Divinity Publishing Ltd *UK* | 2 ratings | Hypnotherapist | £4.99 |
| App name | Platform | Located via script, webpage or both | Main supportive features to support anxiety symptoms in pregnancy | Media | Developer  *Country* | Number of reviews / Installs | Professional input in the App content | Pricing |
| Maternity mindfulness | App store | Script | Mind body: meditation | Audio and written information | Editions La Belle Idee | NR | Not reported | £4.99 |
| MindMum | App store | Script & webpage | Cognitive tools, psychoeducation and mind-body techniques | Written information | Queensland University of Technology  *Australia* | NR | Perinatal mental health professional organisations | Free |
| Mind the bump | App store | Script & webpage | Mind body: mindfulness | Audio and written information | Smiling mind  *Australia* | 31 ratings | Written by psychologists and psychiatrists | Free |
| Mindful mamas | App store | Script & webpage | Mind body: meditation and mindfulness | Audio and written information | Mindful Mamas Club  *US* | 4 ratings | Not reported | $11.99 monthly |
| Peaceful pregnancy: Easy Birth | App store | Script & webpage | Mind body: meditation | Audio and written information | Appiwork PTY Ltd | 2 ratings | Not reported | Free with in-app products |
| Positive pregnancy with Andrew Johnson | App store | Script & webpage | Mind body: meditation | Audio and written information | Universal relaxation *UK* | 4 ratings | Not reported | £2.99 |
| Silatha: Self-love & Meditation for women and mums | App store | Script & webpage | Mind body: meditation | Audio and written information | Silatha: Mindfulness, Balance, Selflove Meditation *UK* | 5 ratings | Not reported | Free with in-app purchases |
| SO-FAR | App store | Script & webpage | Psychoeducation | Audio and written information | Progress IT solutions | NR | Not reported | Free |
| Well-being for pregnancy | App store | Script | Mind body: meditation and relaxation | Audio and written information | The Well-being Series Company SNC | NR | Not reported | Free with in-app products |
