# Supplementary material for: Review of Mobile Apps for Women With Anxiety in Pregnancy: Maternity Care Professionals’ Guide to Locating and Assessing Anxiety Apps
Source: J Med Internet Res. 2022 Mar 23;24(3):e31831. doi: 10.2196/31831 (PMC8987965; doi:10.2196/31831)
Supplement: Multimedia Appendix 4 [file jmir_v24i3e31831_app4.docx]

**Multimedia Appendix 4.** Evaluation criteria for pregnancy anxiety apps (adapted from Van Singer et al [42] and Nouri et al [18]).

| Evaluation criteria for Pregnancy Anxiety Apps | | | |
| --- | --- | --- | --- |
| How do I know if I have anxiety? | Information is present and tailored to pregnancy = 2 | General anxiety symptoms Information is present = 1 | Information is absent = 0 |
| 1. Psychoeducation: App presents comprehensive information about the symptoms of anxiety |  |  |  |
| How do I know the severity of my anxiety symptoms? | Access to validated self-assessment tools with advice or signposting =2 | Access to validated self-assessment tools =1 | Information is absent = 0 |
| 2. App provides access to anxiety self-assessment tools |  |  |  |
| Where can I go for help? | Advice about when and how to contact midwife, GP or other support with contact information and advice = 2 | Advice about when and how to contact midwife, GP or other support = 1 | Information is absent = 0 |
| 3. App includes advice about when, who and how to access support |  |  |  |
| How can I access specialist help? | Information about supportive Perinatal Mental Health Services with referral pathways and contact information =2 | Information about supportive Perinatal Mental Health Services = 1 | Information is absent = 0 |
| 4. App includes information about supportive Perinatal Mental Health Services pregnant women can access |  |  |  |
| What are the treatment options for anxiety? | Comprehensive evidence-based information about therapeutic or supportive care options = 2 | Information about therapeutic or supportive care with little or no reference to the evidence =1 | Information is absent = 0 |
| 5. App provides women with an overview of therapeutic or supportive treatment for anxiety in pregnancy |  |  |  |
| What can I do to help my anxiety symptoms? | Evidence-based therapeutic or coping strategies for anxiety in the context of pregnancy =2 | Evidence-based therapeutic or coping strategies for anxiety =1 | Information is absent = 0 |
| 6. App presents a single or multiple evidence-based coping strategies for anxiety in pregnancy |  |  |  |
| Design and useability | | | |
|  | Yes = 2 | Partly / to some extent = 1 | No = 0 |
| 7. Is the App easy to read and well organised? |  |  |  |
| 8. Is the App is available in other languages? |  |  |  |
| 9. Is the easy App to use? |  |  |  |
| Information and content | | |  |
| 10. Are sources of information included with distinction between scientific and non-scientific content? |  |  |  |
| 11. Is the information in the App objective and free from bias (not selling additional features / products)? |  |  |  |
| 12. Has the App been trialled or tested and reported in published literature? |  |  |  |
| Security and privacy | | | |
| 13. Does the App provide clear security and privacy policies related to personal information? |  |  |  |
| Evidence of maintenance and updating | In the past 6 months =2 | In the past 12 months =1 | Not updated in the past 12 months = 0 |
| 14. Has the App been recently updated in the past 6 months? |  |  |  |
